# Evolution of endoglucanase genes in subterranean and surface isopod crustaceans from Central Western Australia

**DOI:** 10.1002/ece3.10552

**Published:** 2023-09-29

**Authors:** Mohammad Javidkar, Steven J. B. Cooper, Nahid Shokri Bousjein, William F. Humphreys, Rachael A. King, Andrew D. Austin

**Affiliations:** ^1^ Department of Ecology and Evolutionary Biology, School of Biological Sciences, and The Environment Institute University of Adelaide Adelaide South Australia Australia; ^2^ The Brown Foundation Institute of Molecular Medicine The University of Texas Health Science Center Houston Texas USA; ^3^ South Australian Museum Adelaide South Australia Australia; ^4^ Faculty of Biological Sciences Flinders University Bedford Park South Australia Australia; ^5^ Western Australian Museum Welshpool Western Australia Australia; ^6^ School of Animal Biology University of Western Australia Crawley Western Australia Australia

**Keywords:** calcrete aquifers, carbohydrates, lignocellulose, oniscidea

## Abstract

Recent studies have identified a significant number of endogenous cellulase genes in various arthropods, including isopods, allowing them to process hydrocarbons efficiently as a food source. While this research has provided insight into underlying gene‐level processes in cellulose decomposition by arthropods, little is known about the existence and expression of cellulase genes in species from cave environments where carbohydrates are sparse. To investigate whether endogenous cellulase genes are maintained in subterranean species, we sequenced the transcriptomes of two subterranean paraplatyarthrid isopod species from calcrete (carbonate) aquifers of central Western Australia and a related surface isopod species. Seven protein‐coding open‐reading frames associated with endoglucanase genes were identified in all species. Orthology inference analyses, using a wide range of cellulase sequences from available databases, supported the endogenous origin of the putative endoglucanase genes. Selection analyses revealed that these genes are primarily subject to purifying selection in most of the sites for both surface and subterranean isopod species, indicating that they are likely to encode functional peptides. Furthermore, evolutionary branch models supported the hypothesis of an adaptive shift in selective pressure acting on the subterranean lineages compared with the ancestral lineage and surface species. Branch‐site models also revealed a few amino acid sites on the subterranean branches to be under positive selection, suggesting the acquisition of novel adaptations to the subterranean environments. These findings also imply that hydrocarbons exist in subsurface aquifers, albeit at reduced levels, and have been utilized by subterranean isopods as a source of energy for millions of years.

## Graphical abstract

Little is known about the existence and expression of cellulase genes in species from cave environments where carbohydrates are sparse. The current research identified several cellulase genes with endogenous origins from subterranean isopods of Central Western Australia. Furthermore, branch‐site models showed evidence of positive selection acting on a few amino acid sites on the subterranean branches.

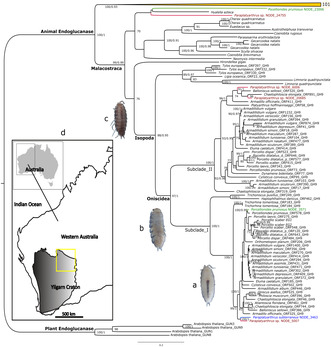

## 1 INTRODUCTION

Subterranean realms are renowned for their aphotic and low variability of abiotic conditions (e.g., temperature, humidity) and are also known for being energy‐limited (oligotrophic) and reliant on the photosynthetic surface environment. As a result, subterranean species are not only characterized by troglomorphies such as reduced or loss of eyes and body pigmentation due to regressive evolution but also the acquisition of special behavioral and physiological features to cope with oligotrophic environments through adaptive processes. Food scarcity in subterranean environments is considered a significant selective force in the evolution of adaptive features in subterranean animals such as smaller body size, higher food utilization efficiency, and reduction in growth rate, metabolic rate, and energy demand (Culver & Pipan, 2009; Hüppop, 2012). Exogenic organic matter (OM), including cellulose produced by plants and phototrophic bacteria, is regarded as the main source of energy to subterranean environments from surface aquatic and terrestrial zones by water flow (Humphreys, 2006).

In addition to the temporo‐spatial limitation of food distribution in hypogean environments (though not all are), the ability of organisms to break down OM is highly variable. It has long been assumed that metazoans can only degrade cellulose via cellulase enzymes derived from their symbiotic protozoa and bacteria in their digestive system. Recent research has provided increasing evidence for the presence of endogenous cellulase enzymes in various arthropods including crustaceans (Bredon et al., 2018; Kostanjsek et al., 2010). For instance, Bredon et al. (2019) using transcriptome data from various marine, freshwater, and terrestrial isopods, identified 40 endogenous lignocellulose degrading CAZymes, with some cellulase and hemicellulase genes found in more than 95% of transcriptomes. Unlike the identification of endoglucanase genes (Glycoside hydrolase family 9 (GH9), subgroup E_2_) in many arthropods, the endogenous exoglucanases (GH7) in eukaryotes and prokaryotes are uncommon (Lombard et al., 2014) and have been reported in a few crustacean species. To the best of our knowledge, research on isopod cellulases has been largely concentrated on the digestive tract and hepatopancreas. However, studies of termites have shown that cephalic tissues are likely to provide an important source of cellulases (Watanabe et al., 1997).

**FIGURE 1:**
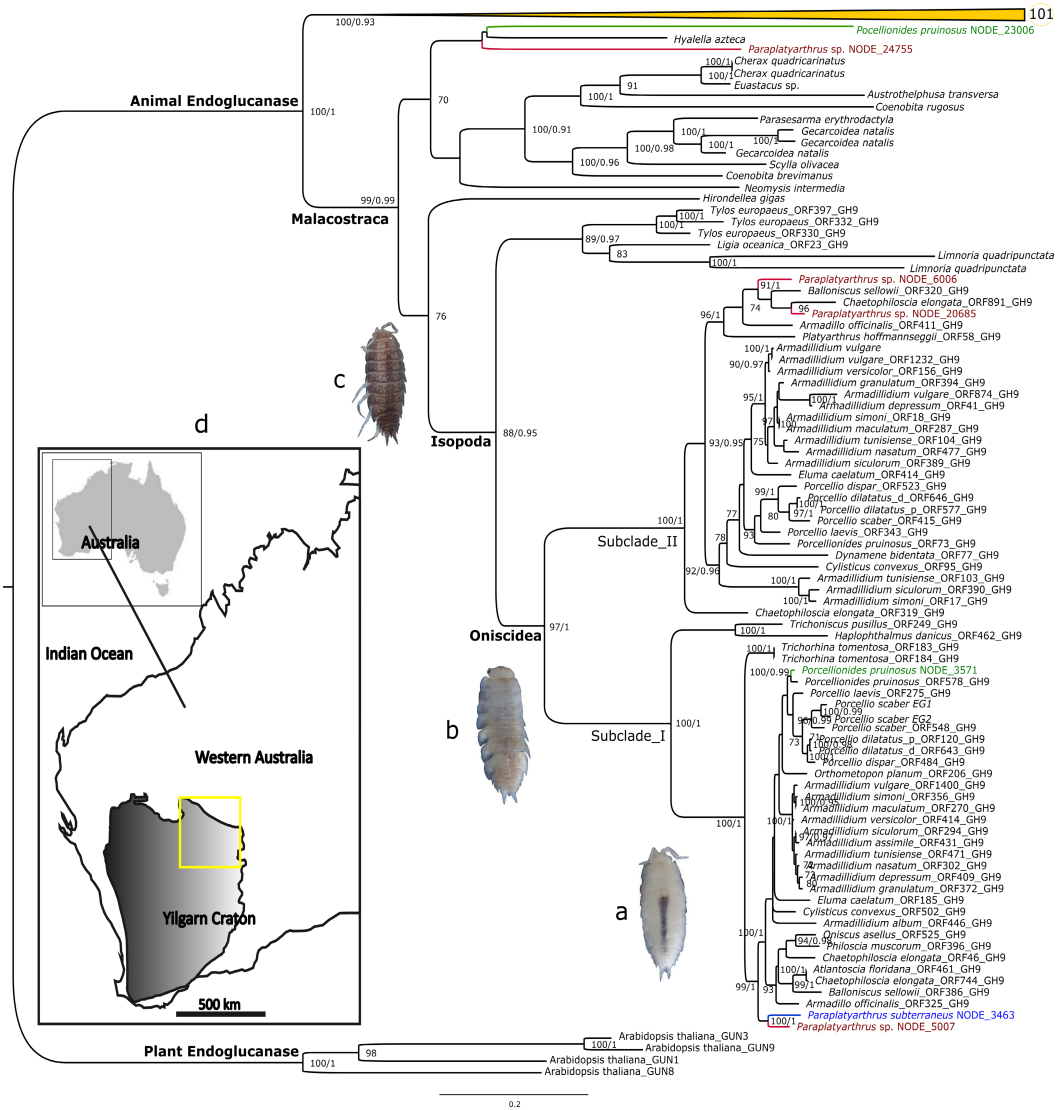
Maximum likelihood protein phylogeny of GH9‐endoglucanase across animal and plant species. Numbers next to the nodes are ML bootstrap and BI posterior probability values, respectively. ML and BI support values less than 70 and 0.95, respectively, are not shown. Branches color‐coded with “red”, “green,” and “blue” represent *Paraplatyarthrus* sp. (troglophile), *Porcellionides pruinosus* (surface), and *Paraplatyarthrus subterraneus* (troglobite) respectively. The collapsed clade shown in yellow comprises other arthropods, some annelid and mollusk species; the circle next to the collapsed clade indicates the number of terminal branches. Images (a–c) show typical troglobite, troglophile, and surface species, respectively. (d) The Yilgarn region of Western Australia; the yellow square indicates the location of calcrete aquifers subjected to sampling.

Although the current evidence has shed light on the ability of arthropods to secrete endogenous cellulases, information on the type and origin of these enzymes in subterranean arthropods, which live in habitats where the organic supply is scarce, is limited to a few subterranean termites (Zhang et al., 2011). The arid central Western Australia (WA), which contains hundreds of isolated groundwater calcrete bodies, harbors a high diversity of subterranean invertebrates, occurring both in and above the aquifer, with species generally confined to individual calcrete bodies (Cooper et al., 2002, 2007, 2008; Guzik et al., 2008; Figure 1). Despite extensive research on the biodiversity and evolutionary history of this subterranean fauna, knowledge of organic supply to these subsurface habitats remains incomplete. Saccò et al. (2022), using radiocarbon fingerprinting and metabarcoding of the gut contents of stygobiotic crustaceans occurring in the subterranean aquifers of the Yilgarn region of WA (Sturt Meadows), reported evidence of organic matter flow to the aquifers from surface ecosystems, implying a link between the subterranean and above‐ground environments. The mean concentrations of dissolved organic carbon in the same region range from 0.39 to 1.94 mg/L (Saccò et al., 2020) during low and high rainfall regimes, respectively, which is in the range of oligotrophic groundwater systems (0.2 to 2 mg/L; Thurman & Malcolm, 1981).

To determine the existence and expression of endogenous cellulase genes in subterranean isopods and their evolution, we examined subterranean isopods in an oligotrophic subterranean ecosystem associated with the Yilgarn calcrete aquifers. The primary aim of our study was to identify putative endogenous endo‐beta‐1,4‐glucanase (endoglucanase) genes involved in cellulolytic activities in two subterranean oniscidean isopods (*Paraplatyarthrus subterraneaus* and *Paraplatyarthrus* sp.) and one surface species, *Porcellionides pruinosus*, using transcriptome data from cephalon tissues. *Paraplatyarthrus subterraneaus* possesses well‐defined troglomorphies (e.g., lack of eyes, no pigmentation) with evidence for genetic isolation within the calcretes (Javidkar et al., 2016) over a long evolutionary time scale. Therefore, it represents a troglobiotic species with populations strongly bound to subterranean habitats. In contrast, *Paraplatyarthrus* sp., with partial eyes, a semi‐pigmented body, and molecular evidence for dispersal between calcretes, is likely to be a troglophile (subterranean species able to live and reproduce underground as well as in the surface environment). Hence, we anticipate that the long evolutionary history of these subterranean isopods in a potentially low‐food environment should promote the acquisition of adaptive features at the amino acid level to efficiently use cellulose as an energy source.

## 2 MATERIALS AND METHODS

### 2.1 Fieldwork

The specimens of *P. subterraneus* (*n*: 7), *Paraplatyarthrus* sp. (*n*: 12), and *P. pruinosus* (*n*: 2) were collected from the Laverton Downs calcrete aquifer (Yilgarn Region, Western Australia; Figure 1). More specifically, the subterranean isopod specimens were sampled from Laverton Downs Windarra calcrete aquifers (S28.4989, E122.1798) using leaf litter traps. The traps were left underground and suspended above the water table for 3–12 months to be colonized by invertebrates and the recovery of the traps was carried out 2–3 times per year (between April and October). After retrieving the traps, the contents were examined to identify any live isopod specimens. These specimens were then preserved in the RNAlater solution (Qiagen) to facilitate the extraction of intact RNA for transcriptome analyses. The surface (epigean) isopods were collected by hand under/between crevices of fallen tree branches and preserved in RNAlater (See Javidkar et al., 2016 for more details on the approach).

### 2.2 Laboratory experiments and sequencing

The specimens of *P. subterraneus*, *Paraplatyarthrus* sp., and *Porcellionides pruinosus* were used for RNA extractions. The cephalothorax for each species was dissected in sterile Petri dishes within RNAlater to avoid RNA degradation during handling. The head samples of each species, with identical COI haplotypes verified using PCR experiments and DNA sequencing, were then pooled within sterile/RNAase‐DNAase safe 2 mL vials. Total mRNA was extracted using a QIAGEN RNeasy Plus Micro Kit (www.qiagen.com). To generate full‐length cDNA libraries, the Clontech SMARTER PCR cDNA Synthesis Kit protocol was followed according to the manufacturer's manual (www.clontech.com). The cDNA libraries for each species were sequenced using an Illumina HiSeq 2000 platform with 100 bp paired‐end reads.

### 2.3 Bioinformatics

FastQC v0.10.1 (http://www.bioinformatics.babraham.ac.uk/projects/fastqc/) was used to assess the quality of the Illumina reads. Trimmomatic v0.22 (Lohse et al., 2012) was applied to remove primers, adapters, and other Illumina‐specific sequences from the reads. The de novo transcriptome assembly analyses were carried out using rnaSPAdes (Bankevich et al., 2012). The assembly metrics were calculated using TransRate v1.0.1 (Smith‐Unna et al., 2016) and Samtools v1.7 (Danecek et al., 2021). BUSCO v5.3.1 (Simão et al., 2015) was utilized to assess the completeness of the transcriptome assemblies with single‐copy orthologs, using arthropods' orthologous groups (number of genomes: 90, number of BUSCOs: 1013). To predict protein‐coding open reading frames (ORFs) within transcripts across the species, Transdecoder V5.5.0 (Haas et al., 2013), with at least 100 amino acids long as minimum criteria to avoid increasing the false‐positive discovery rate, was used. The translated ORFs were then searched against an in‐house database of endoglucanase (15,602 seqs), exoglucanase (5182 seqs) protein sequences obtained from UniProtKB and the published isopod CAZymes (37,481 seqs; Bredon et al., 2019), using NCBI BlastP (Johnson et al., 2008) with an *E*‐value cutoff of 1e^−6^. The putative cellulase‐related ORFs (cORFs) were searched against the UniProtKB/Swiss‐Prot non‐redundant protein sequence database using BLASTP for cross‐validation. The cellulase‐related DNA transcripts identified in the three species, as queries, were searched against the combined transcriptome dataset using BlastN (*E*‐value cutoff: 1e^−6^) for detecting potential cellulase pseudogenes and partial cellulase transcripts not picked up by BlastP. dbCAN3 (https://bcb.unl.edu/dbCAN2/index.php; Zheng et al., 2023), which is an automated carbohydrate‐active enzyme and substrate annotation server, was used to determine the family and activity of the identified enzymes.

### 2.4 Phylogenetic‐oriented orthology inference

A dataset of 194 metazoan endoglucanase amino acid sequences was aligned using the MAFFT multiple aligner implemented in Geneious 9.1.4 (https://www.geneious.com). To further verify the identity of putative cellulase orthologs, both Bayesian inference (BI) and maximum likelihood (ML) phylogenetic methods were conducted, using the same amino acid dataset (495 aa), including arthropods, mollusks, and annelids. The endoglucanase sequences of *Arabidopsis thaliana* (plant) were used as the outgroup. WAG + I + G4 (LogL: −38128.063, AICc: 79836.780) was determined to be the best‐fit model of protein evolution using AICc criteria with ModelFinder (Kalyaanamoorthy et al., 2017). The ML analysis (1000 bootstraps) was carried out using IQ‐TREE v1.6.12 (Hoang et al., 2018). The BI analysis was performed using two independent runs, 11 million generations, subsampling trees/parameters every 100 generations, and a 10% burn‐in, as implemented in MrBayes v3.2.0 (Huelsenbeck & Ronquist, 2005). The final standard deviation of split frequencies was 0.009, suggesting convergence had occurred.

### 2.5 Selection pressure

**FIGURE 2:**
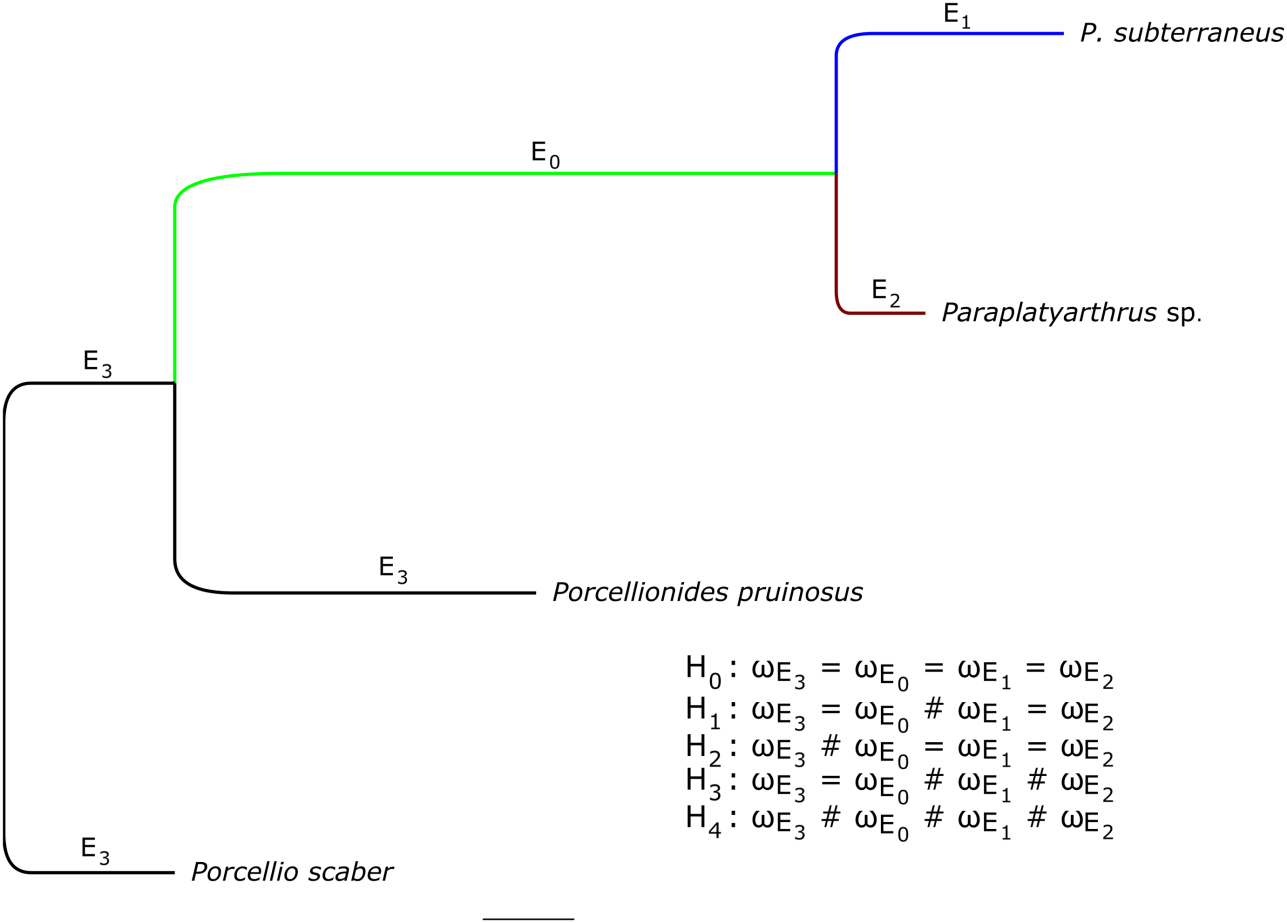
Branch models of *ω* variations across the phylogeny. H_0_–H_4_ represent different hypotheses on the evolutionary trends of selective pressures acting on the ancestral lineage (E_0_), subterranean (E_1_, E_2_), and surface (E_3_) branches.

To investigate potential selective pressures on endoglucanase ORFs (enORFs), using the site (M0 (one‐ratio), M1a (NearlyNeutral), M2a (Selection), M3 (Discrete), M7 (beta), M8 (beta and *ω*)), branch and branch‐site models, PAML v4.9j was used (Yang, 2007). For the branch‐site model (Model A), both Naive Empirical Bayes (NEB) and Bayes Empirical Bayes (BEB) were used for calculating the posterior probability of site classes. Five evolutionary hypotheses to examine potential adaptive trends were investigated using branch models, allowing *ω* (*d*_N_/*d*_S_: nonsynonymous rate to synonymous rate ratio) variations along the target branches (Figure 2): H_0_: Homogenous selective pressure over the phylogenetic tree; H_1_: Homogenous adaptive shift in selective pressure acting on the troglobite (E_1_) and troglophile (E_2_) species different from the ancestral lineage (E_0_, probably a surface ancestor) and surface species (E_3_); H_2_: Homogenous long‐term shift in selective pressure acting on E_0_ and subterranean species (E_1_ & E_2_), different from E_3_; H_3_: Heterogeneous adaptive shift in selection acting on subterranean species with E_1_ and E_2_ subject to differential selective pressures, and different from E_0_/E_3_; H_4_: Heterogeneous selective pressures across most of the phylogeny. Twice the log‐likelihood difference (2∆L = 2(L_1_−L_0_)) between the nested models was compared using a chi‐square (χ^2^) distribution with df = 1 at a 5% significance level.

Furthermore, tests of FEL to detect selection pressure for each site along the entire phylogeny, the branch‐site model, aBSREL, to discover the proportion of branches under positive pressure, BUSTED to evaluate gene‐wide episodic diversifying positive selection, and relaxed selection (RELAXED) were carried out via the datamonkey server (Weaver et al., 2018). For the selection tests, to obtain resolved branching patterns and boost the power of analyses, only canonical enORFs (with at least two conserved domains (Table S2) for the predicted peptides; Davison & Blaxter, 2005) were used. Exoglucanase was excluded from the selection and phylogenetic analyses as it was detected in just one species.

## 3 RESULTS

### 3.1 Assembly, blast search, and orthology inference

Following the de novo transcriptome assembly of the cDNA libraries, a total of 32,245, 30,014, and 30,087 transcripts were generated for *P. subterraneus*, *Paraplatyarthrus* sp., and *P. pruinosus*, respectively (Table S1). Based on the BUSCO analyses, of the total 1013 BUSCO groups searched across the arthropod genomes, 79.6%, 79.3%, and 75.7% complete‐and‐fragmented BUSCOs were recovered for *P. subterraneus*, *Paraplatyarthrus* sp., and *P. pruinosus*, respectively (See Table S1). Based on the BlastP analyses, eight canonical and partial cORFs with significant homology (*E* value < 10^−20^) to endoglucanase and exoglucanase (the latter, just in *Paraplatyarthrus* sp.) were identified (Table 1). No additional cellulase‐related transcripts or pseudogenes were identified using BlastN. According to the BlastP analyses, one, two, and four endoglucanase genes were identified as belonging to the troglobite, surface, and troglophile species, respectively. The sum of per base read depths (the total read base count) for each cORF is also provided in Table 1.

**TABLE 1:** Transcripts associated with endoglucanase and exoglucanase (*) genes in surface (Ω), and subterranean troglobite (‡) and troglophile (†) species, enzyme family (EF), Enzyme Commission (E.C.) number, transcript length (TL), total read base count (i.e., the sum of per base read depths (Bedcov)), ORF length (aa), the estimated molecular weight of the predicted peptides, and accession numbers. Canonical and partial (<150 aa) cORFs are shown with C and P, respectively.

Species	Cellulase_ Transcripts	EF	E.C.	TL (bp)	Bedcov	ORF_length (aa)	C/P	MW (KDa)	Accessions
*Porcellionides pruinosus* ^Ω^	NODE_3571	GH9	3.2.1.4	1306	12,297	412	C	46.00	OR31528
*Paraplatyarthrus subterraneaus* ^‡^	NODE_3463	GH9	3.2.1.4	1494	12,759	481	C	53.36	OR31527
*Paraplatyarthrus* sp. ^†^	NODE_5007	GH9	3.2.1.4	1265	23,509	426	C	47.27	OR31529
*Paraplatyarthrus* sp.^†^	NODE_6006	GH9	3.2.1.4	1123	13,461	356	C	38.67	OR31530
*Paraplatyarthrus* sp.^†^	NODE_20685	–	–	361	2011	119	P	12.73	OR31531
*Paraplatyarthrus* sp.^†^	NODE_24755	–	–	308	392	102	P	11.29	OR31532
*Paraplatyarthrus* sp.^†^*	NODE_18582	–	–	401	1246	133	P	14.12	OR31533
*Porcellionides pruinosus* ^Ω^	NODE_23006	–	–	300	–	100	P	11.29	OR31534

Both the ML and BI phylogenetic analyses generated similar topologies (Figure 1). The putative enORFs were all placed into the monophyletic Malacostraca endoglucanase clade (support values: bootstrap (bp) = 99, posterior probability (pp) = 0.99; Figure 1). The majority of enORFs were placed into a monophyletic oniscidean isopod endoglucanase clade (bp = 97, pp = 1), forming two major subclades (I and II) with all canonical enORFs clustered into Subclade_I (bp = 100, pp = 1). The phylogenetic placements of enORFs within the oniscidean clade with high support values indicate that the endogenous proteins are most likely encoded by the isopod genomes.

### 3.2 Evolutionary selective pressures

To detect possible positive selection associated with amino acid codon sites over the whole phylogeny, multiple tests of selection pressures were carried out. Evidence for positive selection over the whole phylogeny was not detected using the site models (Model comparisons: M1a–M2a and M7–M8) using a likelihood ratio test (LRT) at 95% confidence (Table S3); however, the FEL model showed a single codon under diversifying positive selection (codon 77: LRT = 4.194, *p*‐value = 0.041 < .05). The comparison of M3–M0 models provided strong evidence of heterogeneous selective pressures across the codon sites over the phylogeny (LRT: 31.064, df = 4, χ^2^ *p*‐value = .000).

To assess whether there was evidence of an adaptive shift in selection pressure or positive selection acting on the endoglucanase genes of the subterranean species, branch models were used. For the branch models (Table 2), the H_1_ hypothesis (H_0_ as null) was found to be highly significant (H_0_–H_1_, df = 1, χ^2^ *p*‐value = .000), while the H_2_ model (H_0_ as null) was not supported (H_0_–H_2_, df = 1, χ^2^ *p*‐value = .142). In addition, the H_3_ hypothesis was not favored over H_1_ (H_1_ as null; H_1_–H_3_: df = 1, χ^2^ *p*‐value = .584), with H_4_ also not being supported (H_3_ as null) by the data (H_3_–H_4_, df = 1, χ^2^ *p*‐value = .326). Based on the LRT analyses and AIC criteria, the H_1_ hypothesis (Table 2) was the best‐fit model of *ω* variations along the branches, suggesting there was an adaptive shift in selective pressure acting on the endoglucanase genes of the troglobite and troglophile isopod species. The branch‐site model of positive selection (Model A) was found to be significant at 95% confidence (null model fixes *ω*_2_ = 1; LRT = 4.008, df = 1, χ^2^ *p*‐value = .045). As a result, NEB and BEB detected site classes of positive selection with high posterior probabilities, on a few codon sites, along the subterranean branches (Table 3). Moreover, aBSREL found evidence of episodic diversifying selection for the troglobite branch on the phylogeny (*ω*_1_ = 0.00 (95%), *ω*_2_ = 5.09 (5.1%), LRT = 4.389, *p*‐value = .040; 95% confidence). BUSTED also found evidence of diversifying positive selection for at least one site on at least a test branch for the subterranean branches (lnL = −1825.4, LRT, *p*‐value = .021; Table S4). The tests of relaxed selection were not significant (*p* > .05; Table S5).

**TABLE 2:** Parameter estimates under variable *ω* ratios among branches, log‐likelihood (L) values under different models following ML estimations; the number of free parameters (np), Akaike Information Criterion (AIC) index, and LRTs (2∆L) of branch model comparisons using χ^2^ distribution (df = 1, only nested models were compared).

Branch models and their comparisons	$\omega_{E_{0}}$	$\omega_{E_{1}}$	$\omega_{E_{2}}$	$\omega_{E_{3}}$	L	np	AIC	LRT (2∆L)
H_0_: $\omega_{E_{3}}$ = $\omega_{E_{0}}$ = $\omega_{E_{1}}$ = $\omega_{E_{2}}$	0.050	0.050	0.050	0.050	−1866.285	7	3746.57	–
H_1_: $\omega_{E_{3}}$ = $\omega_{E_{0}}$ ≠ $\omega_{E_{1}}$ = $\omega_{E_{2}}$	0.033	0.165	0.165	0.033	−1857.967	8	3731.934	–
H_2_: $\omega_{E_{3}}$ ≠ $\omega_{E_{0}}$ = $\omega_{E_{1}}$ = $\omega_{E_{2}}$	0.063	0.063	0.063	0.036	−1865.211	8	3746.422	–
H_3_: $\omega_{E_{3}}$ = $\omega_{E_{0}}$ ≠ $\omega_{E_{1}}$ ≠ $\omega_{E_{2}}$	0.032	0.127	1.056	0.032	−1857.534	9	3733.068	–
H_4_: $\omega_{E_{3}}$ ≠ $\omega_{E_{0}}$ ≠ $\omega_{E_{1}}$ ≠ $\omega_{E_{2}}$	0.028	0.125	1.827	0.037	−1857.335	10	3734.67	–
H_0_ vs H_1_	–	–	–	–	–	–		16.636**
H_0_ vs H_2_	–	–	–	–	–	–		2.149
H_1_ vs H_3_	–	–	–	–	–	–		0.351
H_3_ vs H_4_	–	–	–	–	–	–		0.398

*Note*: Two asterisks indicate a very significant LRT (*p* < .01, χ^2^ critical value = 6.63).

**TABLE 3:** Parameters in the branch‐site Model A including the proportion of site classes, *ω* for background (surface species) and foreground (subterranean species) lineages, and positively selected sites (PSS, *ω* > 1) using BEB and NEB methods along the foreground branches.

Site class	Proportion	Background *ω*	Foreground *ω*	PSS using BEB	PSS using NEB
0 (0 < *ω*_0_ < 1)	0.913	0.018	0.018	K* (15), W* (85), W* (91), G (189)	**K** (15), W* (85), W* (91), Q (53), A (72), S (77), L (145), G (189), T (202), T (220), D (248)
1 (*ω*_1_ = 1)	0.036	1.000	1.000		
2a Background (0 < *ω*_0_ < 1) Foreground (*ω*_2_ ≥ 1)	0.049	0.018	4.223		
2b Background (*ω*_2_ = 1) Foreground (*ω*_2_ ≥ 1)	0.002	1.000	4.223		

*Note*: Amino acids with bold font indicate positive sites with posterior probability (*p*) > .99, asterisks show positive sites with *p* > .95, and plain font denotes positive sites with *p* > .5. Site numbers are shown in parentheses.

## 4 DISCUSSION

This study provides the first evidence for the expression of cellulase genes in subterranean isopods and shows their phylogenetic relationships to other known endoglucanase genes in invertebrates. The monophyly of the putative enORFs with the isopods' endogenous endoglucanase orthologs, including the experimentally verified endoglucanase in *Porcellio scaber* (Kostanjsek et al., 2010; Figure 1, Subclade_I), and *Armadilludium vulgare* (Bredon et al., 2018, 2019), supports the endogenous origin of the enORFs in both subterranean and surface isopods analyzed here.

Based on the branch models, there is evidence that the subterranean species were subjected to a homogenous adaptive shift in selective pressure (a 5‐fold change in selection pressure; H_1_ hypothesis, Table 2) compared with the ancestral lineage and surface species. This shift in selective pressure could be linked to the evolutionary transition from surface to subterranean environments. It is estimated that this transition by *Paraplatyarthrus* species in WA took place between the mid‐Miocene and the Pleistocene, coinciding with the onset of aridification in Australia (Javidkar et al., 2018). Furthermore, branch‐site models of evolution (Model A, aBSREL, and BUSTED) provided evidence of positive selection for the subterranean species that may indicate novel adaptations to the subterranean environment at the associated amino acid sites. These findings could also suggest that the acquisition of adaptive features and, as a result, the retention of endoglucanase genes in the subterranean isopods, which have persisted over long evolutionary times in calcrete aquifers, may be a key factor for their long‐term survival in a potentially low‐energy habitat. Although our study detected positive selective signals on the subterranean branches, such signals were not identified for GH9 genes (endo‐β‐1,4‐glucanases (E.C. 3.2.1.4)) in terrestrial isopods (Bredon et al., 2019); the latter found just a single site under positive selection using the program FUBAR.

In contrast to other studies on the expression of cellulase genes in isopods using whole animal bodies, digestive tract, or hepatopancreas, this study utilized head samples to elucidate the likely role of cephalic tissues (e.g., mouth tissues, salivary glands) in the secretion of endogenous cellulolytic enzymes in isopods. The expression of endogenous endoglucanase from salivary glands has also been confirmed in several termite species (e.g., Nakashima et al., 2002). However, to better understand the evolutionary history of the complete set of endoglucanase genes in subterranean isopod species and improve recognition of the role and contribution of different tissues in the expression of cellulase genes, digestive tract, and hepatopancreas tissues also need to be utilized.

The occurrence of multiple enORFs per transcriptome, found for the troglophile (four enORFs) and surface (two enORFs) species in this study, is common in terrestrial isopods (Bredon et al., 2018, 2019). According to Bredon et al. (2019), the number of endogenous cellulase genes (GH9) identified in the digestive tracts of terrestrial isopods ranged from one to 12, reflecting different evolutionary histories. In this study, although the difference in the number of predicted cORFs among the species could be due to the difference in sequencing reads between libraries, based on the BUSCO analyses, the troglobite species, with just a single enORF identified, showed the same or even a higher BUSCO score compared to those of other assemblies (C + F: 79.6%; Table S1). Also, the medium‐level BUSCO scores recovered here may indicate that some cORFs are missing from our prediction analyses. Moreover, considering the different tissue used in this study, a direct quantitative comparison with data from published research is not plausible.

The selection analyses, and previous biogeographic analyses of the subterranean isopods (Javidkar et al., 2018), indirectly suggest that the calcrete aquifers have been fed with lignocellulose hydrocarbons for millions of years. This lignocellulose is likely to come from root systems that penetrate into the calcrete and down to the water table, where they also provide a direct (via root grazing) or indirect (via exudates) source of carbon for other crustaceans, such as Amphipoda and Copepoda, in the groundwater ecosystem; It has been suggested that exudates may be derived from termites in the terrestrial environment (Saccò et al., 2022), but it is also likely that subterranean isopods, by directly feeding on root material, also contribute to energy flows into the groundwater.

## AUTHOR CONTRIBUTIONS

**Mohammad Javidkar:** Conceptualization (lead); data curation (lead); formal analysis (lead); investigation (lead); methodology (lead); resources (lead); software (equal); supervision (equal); validation (equal); visualization (lead); writing – original draft (lead); writing – review and editing (lead). **Steven J. B. Cooper:** Conceptualization (equal); funding acquisition (lead); investigation (supporting); methodology (supporting); project administration (lead); software (supporting); supervision (equal); writing – original draft (supporting); writing – review and editing (equal). **Nahid Shokri Bousjein:** Data curation (supporting); formal analysis (equal); investigation (equal); methodology (equal); software (equal); validation (equal); visualization (equal); writing – review and editing (supporting). **William F. Humphreys:** Conceptualization (supporting); funding acquisition (lead); investigation (supporting); project administration (lead); resources (equal); supervision (supporting); writing – review and editing (supporting). **Rachael A. King:** Conceptualization (supporting); investigation (supporting); supervision (equal); writing – review and editing (supporting). **Andrew D. Austin:** Conceptualization (equal); funding acquisition (lead); investigation (supporting); project administration (lead); supervision (equal); writing – original draft (supporting); writing – review and editing (supporting).

## CONFLICT OF INTEREST STATEMENT

The authors have no conflicts of interest to declare.

## Supporting information


Appendix S1

## Data Availability

All enROF and cORF sequences will be deposited in GenBank upon acceptance (Table 1). The datasets for the phylogenetic inference and selection pressure analyses are provided as Appendix S1.
