# Supplementary material for: Evolution of endoglucanase genes in subterranean and surface isopod crustaceans from Central Western Australia
Source: Ecol Evol. 2023 Sep 29;13(10):e10552. doi: 10.1002/ece3.10552 (PMC10541295; doi:10.1002/ece3.10552)
Supplement: Supplementary file 1 — Appendix S1 [file ECE3-13-e10552-s001.zip › ece310552-sup-0001-TablesS1-S5.docx]

**Table S1-** The statistics include the number of reads, the number of contigs (N_seqs), the mean length of the contigs (Mean_len), the number of contigs shorter than 200 bases (N_under_200), the number of contigs greater than 1,000 bases long (N_over_1K), N50, Complete BUSCOs (C), Fragmented BUSCOs (F), and Missing BUSCOs (M).

| Species | Number of reads | N_seqs | Mean_len | N_ under_ 200 | N_ over_ 1k | N50 | C | F | M |
| --- | --- | --- | --- | --- | --- | --- | --- | --- | --- |
| *Paraplatyarthrus* sp. | 24,795,047 | 32245 | 746.65015 | 219 | 7014 | 1080 | 68.0% | 11.3% | 20.7% |
| *Paraplatyarthrus subterraneaus* | 21,896,830 | 30014 | 752.70417 | 201 | 6611 | 1093 | 68.7% | 10.9% | 20.4% |
| *Porcellionides pruinosus* | 23,941,206 | 30087 | 675.72516 | 208 | 5415 | 920 | 63.4% | 12.3% | 24.3% |

**Table S2-** The alignment of three conserved domains (Davison & Blaxter, 2005) in exemplar arthropods’ endoglucanase (GH9) and the species in this study. Catalytically important conserved residues are shown in bold font. C1 and C2 refer to NODE_5007 and NODE_6006, respectively.

| Species | Conserved Region I | Conserved Region II | Conserved Region III |
| --- | --- | --- | --- |
| *Apis mellifera* (Honey bee) | LTGGYY**D**AG**D**FVKFGFT | PPKQP**H**HAASSCP | ADKFH**D**HREDYVYT**E**VTL |
| *Cherax quadricarinatus* (Crayfish) | LTGGYY**D**AG**D**HVKFGFP | PPTRP**H**HRSSSCP | DGSYN**D**DRQDYQHN**E**VAC |
| *P. subterraneaus* | LVGGYF**D**AG**D**FVKFGFP | PPVRP**H**HRAASCP | NDQYT**D**DRNDYTHN**E**VAD |
| *Paraplatyarthrus* sp./C1 | LVGGYF**D**AG**D**FVKFGFP | PPVRP**H**HRAASCP | NDQFN**D**DRNDYTHN**E**VAD |
| *Paraplatyarthrus* sp./C2 | ??? | PPERP**H**HRSSSCP | SDQYT**D**SRDDYIHN**E**VAC |
| *Porcellionides pruinosus* | LVGGYF**D**AG**D**FVKFGFP | PPVRP**H**HRAASCP | ??? |
| *Porcellio scaber* (Woodlouse) | ??? | PPVRP**H**HRSASCP | DDQFN**D**DRNDYTHN**E**VAT |
| *Nasutitermes takasagoensis* (Termite) | LTGGYF**D**AG**D**FVKFGFP | PPTRP**H**HRSSSCP | NDNYV**D**DRSDYVHN**E**VAT |

**Table S3-** Parameter estimates for site models including dN/dS (**ω**) and proportion (**p**_i_) of ω classes; parameters in parentheses are not free; **np** denotes the number of free parameters; **L** indicates log-likelihood values for each model. p & q in M7/M8 models show the shape and scale parameters of the beta distribution for ω classes (0≤ ω≤1), respectively.

| Model | np | L | Parameters |
| --- | --- | --- | --- |
| M0 | 1 | -1816.084 | ω= 0.044 |
| M1a | 2 | -1802.746 | ω_0_ = 0.02, (ω_1_ = 1)  p_0_ = 0.935, (p_1_= 0.064) |
| M2a | 4 | -1802.746 | ω_0_ = 0.02, (ω_1_ = 1), ω_2_ =?  p_0_ = 0.935, p_1_= 0.064, (p_2_= 0.000) |
| M3 ( k=3) | 5 | -1800.552 | ω_0_ = 0.003, ω_1_ = 0.003, ω_2_ = 0.336  p_0_ = 0.251, p_1_= 0.58, (p_2_=0.17) |
| M7 (beta) | 2 | -1800.644 | p = 0.087, q = 1.225 |
| M8 (beta&ω>1) | 4 | -1800.644 | p = 0.087, q = 1.225  p_0_ = 0.999, (p_1_= 0.000), ω_1_= 1.000 |

**Table S4-** BUSTED test of gene-wide episodic diversifying selection for the subterranean branches of the phylogeny (LRT, p-value = 0.021 ≤ .05)

| Model | Log L | np | AIC_c_ | Branch test | ω_1_ | ω_2_ | ω_3_ |
| --- | --- | --- | --- | --- | --- | --- | --- |
| Unconstrained | -1825.4 | 34 | 3721.2 | *Test (subterranean branches)* | 0.02  (55.63%) | 0.02  (41.29%) | 10.64  (3.08%) |
|  |  |  |  | *Background (surface species)* | 0.01  (0.73%) | 0.01  (97.02%) | 2.15  (2.25%) |

**Table S5-** Tests of Relaxed Selection using RELAX for evolutionary branches of the endoglucanase gene: Subterranean species (**S**: Troglobite & Troglophile), Troglobite (**Tb**), Troglophile (**Tp**), Ancestral lineage to subterranean species (**Al**) and the epigean species, *p. pruinosus* (**E**).

| Test Branches | Reference Branches | Intensity Parameter (K) | Likelihood Ratio (LR) | P-value |
| --- | --- | --- | --- | --- |
| S | Al | 0.37 | 1.00 | 0.317 |
| S | E | 2.56 | 1.21 | 0.272 |
| S+Al | E | 0.62 | 1.19 | 0.275 |
| Tb | Al | 0.26 | 0.67 | 0.414 |
| Tp | Al | 0.00 | 1.55 | 0.213 |
